# Facile Preparation of Self-Assembled Polydopamine-Modified Electrospun Fibers for Highly Effective Removal of Organic Dyes

**DOI:** 10.3390/nano9010116

**Published:** 2019-01-18

**Authors:** Cuiru Wang, Juanjuan Yin, Ran Wang, Tifeng Jiao, Haiming Huang, Jingxin Zhou, Lexin Zhang, Qiuming Peng

**Affiliations:** 1 State Key Laboratory of Metastable Materials Science and Technology, Yanshan University, Qinhuangdao 066004, China; wcr2016ysdx@163.com; 2 School of Environment and Civil Engineering, Dongguan University of Technology, Dongguan 523808, China; huanghaiming52hu@163.com; 3 Hebei Key Laboratory of Applied Chemistry, School of Environmental and Chemical Engineering, Yanshan University, Qinhuangdao 066004, China; jjy1729@163.com (J.Y.); wr1422520780@163.com (R.W.); zhoujingxin@ysu.edu.cn (J.Z.); zhanglexin@ysu.edu.cn (L.Z.)

**Keywords:** electrospinning, solvent vapor annealing, structural regularity, polydopamine, dye removal

## Abstract

Polydopamine (PDA) nanoparticles can be used as an adsorbent with excellent adsorption capacity. However, nanosized adsorbents are prone to aggregation and thus are severely limited in the field of adsorption. In order to solve this problem, we utilized polydopamine in-situ oxidation self-polymerization on the surface of polycaprolactone (PCL)/polyethylene oxide (PEO) electrospun fiber after solvent vapor annealing (SVA) treatment, and successfully designed and prepared a PCL/PEO@PDA composite membrane. The SVA treatment regulated the microscopic morphology of smooth PCL/PEO electrospun fibers that exhibited a pleated microstructure, increasing the specific surface area, and providing abundant active sites for the anchoring of PDA nanoparticles. The PCL/PEO@PDA composite obtained by chemical modification of PDA demonstrated numerous active sites for the adsorption of methylene (MB) and methyl orange (MO). In addition, the PCL/PEO@PDA composites were reusable several times with good reutilization as adsorbents. Therefore, we have developed a highly efficient and non-agglomerated dye adsorbent that exhibits potential large-scale application in dye removal and wastewater purification.

## 1. Introduction

In recent years, the rapid development of industrialization has caused serious water pollution problems, which have received widespread attention [1,2]. Among the various pollutants, organic dyes are one of the main sources of water pollution. Therefore, finding a convenient method for effectively removing organic dyes from wastewater is a serious challenge [3,4,5]. A variety of physical, chemical, and biological techniques have been reported for the removal of dyes from wastewater including adsorption, coacervation, membrane separation, etc. [6]. Adsorption is an effective and economical method for purifying dye wastewater due to its high efficiency, simplicity of operation, and insensitivity to contaminants [7,8]. Various adsorbents such as natural materials, activated carbon, hydrotalcite, nano-oxide particles, and biological adsorbents are used for wastewater purification [9,10]. Due to its simple preparation method, high porosity, large specific surface area, and high adsorption efficiency, nano-adsorbents exhibit significant adsorption capacity [11]. For instance, Luo et al. [12] utilized a submerged circulation impinging stream reactor (SCISR) to prepare a millimeter-sized cellulose bead adsorbent named MCB-AC, which was applied to wastewater treatment. However, some nano-adsorbents were prone to agglomeration and were difficult to regenerate, thus severely limiting their use in the field of adsorption [13]. Consequently, it is of great significance to find organic dye adsorbents with high adsorption capacity and recyclability.

Electrospinning technology has become one of the most effective methods for preparing fibers due to its obvious advantages of simple operation and easy regulation [14,15]. At present, the construction of nanostructures of various materials such as polymers, inorganic substances, and multi-component composites has been realized by electrospinning technology, which simultaneously regulates the size and morphology of the nanostructures [16,17,18]. Electrospun fibers have the advantages of large specific surface area, porosity, and excellent flexibility, showing great potential in the fields of catalysis, drug carriers, and ultrafiltration [19,20]. In addition, the fiber material exhibited good adsorption capacity due to its microporous structure. For example, Miao et al. [21] combined electrospinning technology and a hydrothermal reaction to prepare SiO_2_@ALOOH core/shell fiber and applied it to wastewater purification. Obviously, the electrospun fiber membrane met the stringent requirements in the field of decontaminating wastewater. It should be noted that polydopamine (PDA) has been reported as an effective adsorbent [22,23]. The polymer’s abundant reactive functional groups such as amino, imino, and phenolic hydroxyl groups seemed beneficial for secondary reactions with other ions. In addition, PDA-coated nanomaterials increased the numerous active sites and improved the adsorption performance. Dong et al. [23] prepared a PDA-coated graphene oxide (GO/PDA) composite adsorbent using the synergistic effect of PDA and graphene oxide that showed a higher adsorption capacity than pure GO and PDA. Polycaprolactone (PCL), which has good biocompatibility and degradability, could be used as an electrospinning material. Furthermore, the addition of a polyethylene oxide (PEO) component with abundant oxygen-containing groups was proposed to regulate the phase structures and interfacial active sites at suitable conditions. It has been reported that the PCL/PEO fibers prepared by electrospinning carried considerable free amorphous PCL chains [24,25,26]. During solvent vapor annealing (SVA) treatment, the free amorphous PCL chains absorbed acetone vapor faster than the chains in the crystallization zone. However, PEO, playing the role of mini dividers, limited the growth of semi-crystalline PCL. Thus, the swollen amorphous PCL chains were deposited on the crystalline lamellae of preexisting PCL or PEO while the PEO phase remained largely unchanged. Once the desiccator of the SVA treatment was turned on, the acetone vapor quickly left the fiber system, creating an obvious morphological change due to the amorphous chains that preferentially crystallized on the edge of the pre-existing crystallites [25].

In order to solve the abovementioned difficulties, we utilized the in-situ oxidation self-polymerization of polydopamine by the good adsorption capacity of electrospun fibers and polydopamine by using electrospun polycaprolactone (PCL)/polyethylene oxide (PEO) as the substrate. This was combined with the SVA method to regulate the surface morphology of the substrate, and we successfully designed and prepared a PCL/PEO@PDA composite adsorbent. The SVA treatment controlled the layered structures, which increased the specific surface area to provide more active sites for the PDA coating. It was found that the prepared PCL/PEO@PDA composite showed excellent dye adsorption capacity due to its wrinkled morphology and improved hydrophilicity via the PDA layer. In addition, the obtained composite adsorbents could be utilized several times with good stability and recyclability, demonstrating wide applications in wastewater treatment and composite materials.

## 2. Experimental Method

### 2.1. Materials

Both polycaprolactone (PCL, Average Mn = 80,000) and polyethylene oxide (PEO, average Mv = 600,000) were purchased from Sigma–Aldrich. Chloroform (analytical grade, 99.0%) and acetone (analytical grade, 99.5%) were obtained from Tianzheng Chemical Reagent (Tianjin, China). Tris (hydroxymethyl) aminomethane hydrochloride (Tris HCL, super purity, 99%, Aladdin), sodium hydroxide (NaOH, AR, Kermel Reagent, Tianjin, China), dopamine hydrochloride (98%, Aladdin), methylene blue (MB), methyl orange (MO), Safranine T (ST), and Rhodamine B (RhB) were from Aladdin without further purification. All aqueous solutions were prepared using ultrapure water throughout the experiment. All chemicals were used as received.

### 2.2. Preparation of PCL/PEO@PDA Nanocomposites

In our previous work, PCL and PEO were simultaneously dissolved in chloroform, and magnetically stirred overnight at room temperature to obtain a uniform electrospinning precursor solution for the preparation of electrospun fibers [24]. The electrospinning parameters were 15 kV voltage, 1 mL/h flow rate, and 25 cm between the needle and the receiving plate. The prepared PCL/PEO composite fiber was directly separated from the receiving plate and placed in a hermetical desiccator room containing 200 mL acetone at room temperature for five days to optimize the surface morphology. Subsequently, polydopamine modification was performed. Specifically, a 10 mM Tris-HCl buffer solution was prepared to which NaOH was added to adjust the pH to 8.5. Then, dopamine (DA) was added to the above pH-adjusted buffer solution to obtain 2.0 mg/mL of dopamine aqueous solution. Once DA was added, the color of the solution changed from colorless to dark brown in an instant, which meant the formation of polydopamine (PDA) and was similar to previous reports [27,28]. The SVA-treated PCL/PEO was immersed into the aqueous dopamine solution and stirred for different times (5 h, 30 h, and 45 h) to gain PCL/PEO@PDA composite films with different PDA modification time intervals. The PCL/PEO@PDA composite membranes were removed from the aqueous dopamine solution and washed several times with ultrapure water to remove free polydopamine molecules. Finally, it was dried in a vacuum oven at room temperature for 24 h and stored for subsequent use.

### 2.3. Adsorption Capacity Test

The adsorption activity of PCL/PEO@PDA composite membranes to methylene blue (MB) and methyl orange (MO) dyes was measured by a 721 visible spectrophotometer (Shanghai Yidian Analytical Instrument Co. Ltd., Shanghai, China). The entire adsorption experiment was carried out by magnetic stirring at room temperature (298 K). First, four concentration-absorbance calibration curves were established by measuring the absorbance of MB (662 nm), MO (463 nm), RhB (554 nm), and ST (518 nm) solutions at different concentrations according to previous reports [29]. Subsequently, 15 mg of the prepared PCL/PEO@PDA composites as adsorbent materials were added to 50 mL of dye solutions (MB, 10 mg/L; MO, 50 mg/L; RhB, 5 mg/L; and ST, 30 mg/L), respectively. The absorbance of the dye solution was measured at different intervals until the absorbance stabilized. The concentration of the dye solution was calculated from the calibration curve established above. The SVA-treated PCL/PEO composite fiber was used as a reference for the adsorption experiments. Finally, the PCL/PEO@PDA composite adsorbent under optimal PDA modification time was subjected to recycling experiments. A total of 15 mg of the prepared PCL/PEO@PDA was added to a freshly prepared MO (100 mg/L) solution for the adsorption experiments; after reaching adsorption saturation, the PCL/PEO@PDA adsorbent was removed directly and washed several times with ultrapure water and ethanol to further adsorb another fresh MO (50 mg/L) solution. The above experimental procedure was repeated eight times to complete the cyclic adsorption test.

### 2.4. Characterization

The morphologies of the samples were examined via a scanning electron microscope (SEM, FEI Corporate, Hillsboro, OR, USA) with gold plasma deposition. Fourier transform infrared spectroscopy (Thermo Nicolet Corporation) was performed by the KBr pellet method. The thermal stability of the as-prepared samples was investigated by thermogravimetry-differential scanning calorimetry (TG-DSC) under an argon atmosphere using a 409 PC Luxxsi thermal analysis instrument (Netzsch Instruments Manufacturing Co., Ltd., Seligenstadt, Germany). We obtained X-ray photoelectron spectroscopy (XPS) data by monitoring a Thermo Scientific ESCALab 250Xi (Netzsch Instruments Manufacturing Co., Ltd., Seligenstadt, Germany) equipped with 200 W of monochromatic AlKα radiation. The 500 μm X-ray spot was used for XPS analysis. The base pressure in the analysis chamber was about 3 × 10^−10^ mbar. Typically, the hydrocarbon C1s line at 284.8 eV from adventitious carbon is used for energy referencing. Both the survey scan and individual high-resolution scan were recorded. The N_2_ adsorption–desorption properties were measured at 77.3 K by a Quadrasorb analyzer (Quantachrome Instruments, Boynton Beach, FL, USA). Before the measurement, the samples were outgassed at 40 °C under vacuum for 4 h. The surface areas were calculated by the Brunauer–Emmett–Teller (BET) method.

## 3. Results and Discussion

### 3.1. Characterization of PCL/PEO@PDA Composite Adsorbents

**Figure 1:**
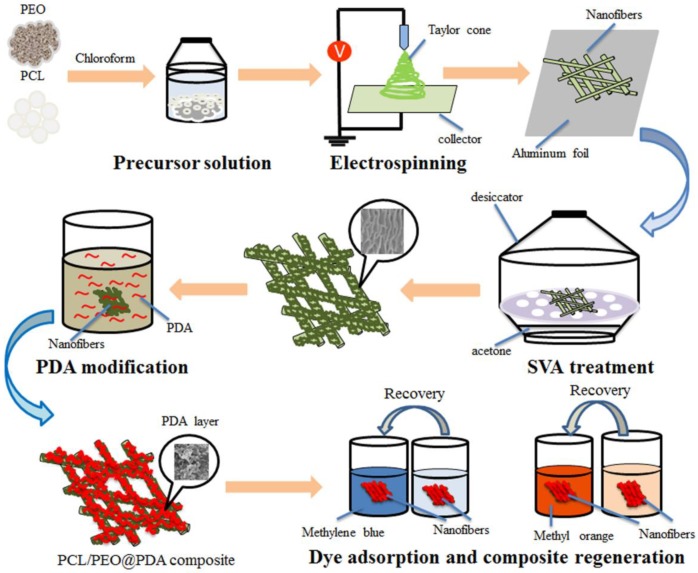
Schematic illustration of the preparation and adsorption dye process of the polycaprolactone/polyethylene oxide@polydopamine (PCL/PEO@PDA) composites as dye absorbents.

**Figure 2:**
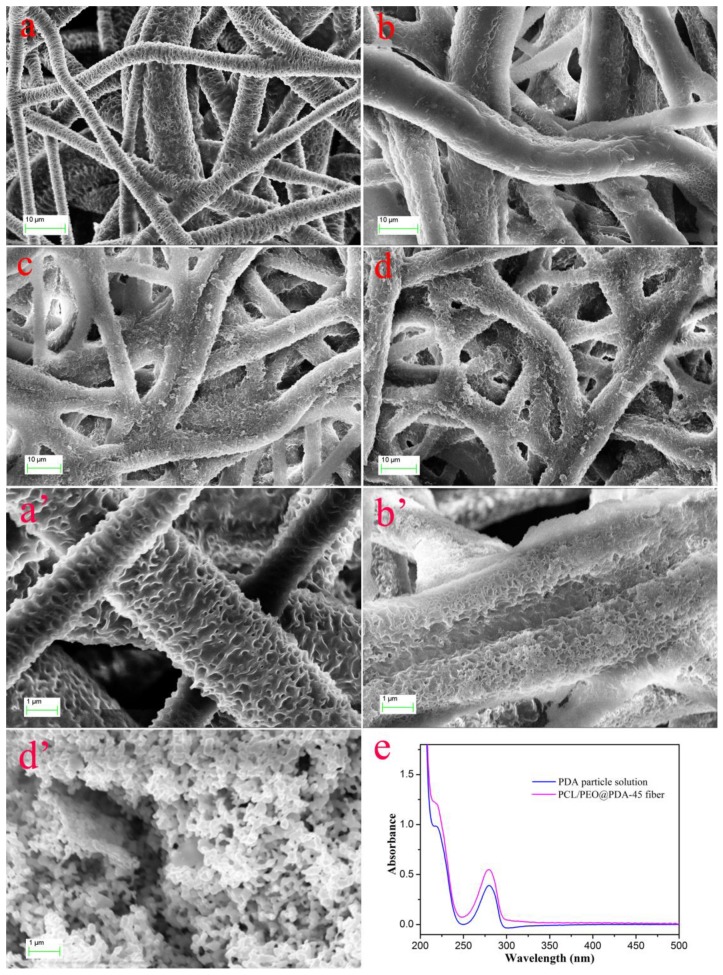
SEM images of the solvent vapor annealing (SVA)-treated PCL/PEO fibers (**a**,**a’**) and PCL/PEO@PDA of different modification time intervals (**b**,**b’**), 5 h; (**c**), 30 h; (**d**,**d’**), 45 h; (**e**) UV-vis spectra of the PDA solution and PCL/PEO@PDA-45 fiber composite.

Figure 1 illustrates the preparation and dye adsorption process of PCL/PEO@PDA as a dye adsorbent. The experimental process was mainly divided into four parts: electrospinning, SVA treatment, chemical modification, and dye adsorption. The electrospinning PCL/PEO fiber membrane was prepared according to the previous work and subjected to subsequent SVA treatment. The SVA-treated PCL/PEO fiber membrane was then chemically modified with polydopamine to obtain the PCL/PEO@PDA dye adsorbent. Finally, the prepared PCL/PEO@PDA composite materials were used as dye adsorbents to adsorb several dyes such as methylene blue and methyl orange. The SEM images of the SVA-treated PCL/PEO and PCL/PEO@PDA with different chemical modification times (5 h, 30 h, and 45 h) are shown in Figure 2. Our previous work reported significant changes in the morphology of electrospun PCL/PEO fiber membranes before and after SVA treatment [24]. The as-prepared electrospun PCL/PEO fiber demonstrated a straight and smooth structure, as shown in our previous report [24]. It was interesting to note that the SVA-treated fiber exhibited a curved and wrinkled structure, as shown in Figure 2a,a’. Figure 2b–d clearly illustrated the differences in the surface morphologies of PCL/PEO@PDA for different PDA modification intervals. For a modification time of 5 h, as shown in Figure 2b,b’, it was obvious that the surface of the wrinkled structure was partially covered by a PDA layer. As the PDA modification time increased, numerous PDA nanoparticles anchored and accumulated on the surface of the wrinkled PCL/PEO composite fiber, which can be clearly seen in Figure 2d,d’. At the same time, as shown in Figure 2e, the UV-vis spectra of the PDA solution and PCL/PEO@PDA-45 fiber composite showed the same peaks at 218 and 280 nm, indicating the formation of PDA particles due to the DA oxidation and the polymerization reaction process [30]. Previous studies have shown that the adhesion behavior of PDA nanoparticles on the surface of solid membranes is mainly due to the noncovalent interactions (such as hydrogen bonding and electrostatic forces) between the hydrophilic amino-groups of PDA and the functional active groups on the surface of the composites [31,32,33,34]. The results of the above SEM topography strongly demonstrate the successful preparation of PCL/PEO@PDA composites. The modification of PDA could improve the hydrophilicity, stability, and adsorption capacity of the prepared composite.

**Figure 3:**
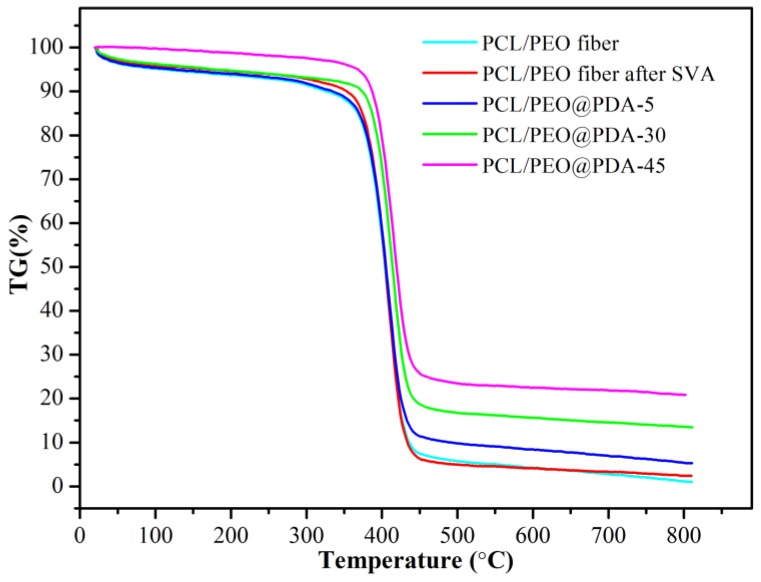
Thermogravimetry (TG) curves of the prepared PCL/PEO electrospun fibers before and after SVA treatment and the PCL/PEO@PDA composites of different PDA modification intervals (5 h, 30 h, and 45 h).

We surveyed the thermogravimetric (TG) curves of the initial samples and subsequent PDA modified samples with different reaction time intervals to investigate the thermal stability of the PCL/PEO@PDA composites. As shown in Figure 3, all samples exhibited a slow weight loss below 250 °C, which corresponded to the removal of the moisture remaining in the samples [35]. In addition, the weight of all samples tended to be stable above 550 °C. The PCL/PEO composite fibers before and after SVA treatment exhibited similar thermal stability, and the qualities’ retention ratios were 1.0% and 2.5%, respectively. The qualities’ retention ratios of the above two samples were close to zero because the PCL and PEO polymers were thermally decomposed at high temperatures into volatiles such as H_2_O, CO_2_, etc. [36]. A sharp weight loss between 380 °C and 440 °C originated from the thermal decomposition of the alkyl chains and various functional groups in these samples [37]. It was reported that pure PDA demonstrated an approximate 62% weight retention ratio at 550 °C [30]. For the present synthesized composite fiber materials, as the chemical modification time increased, the weight retention ratios reached 22% for the PCL/PEO@PDA-45 composite. The decreased weight loss of the PCL/PEO@PDA composites after SVA treatment indicated that substantial PDA nanoparticles had successfully anchored on the surface of the SVA-treated PCL/PEO fibers and significantly improved stability.

**Figure 4:**
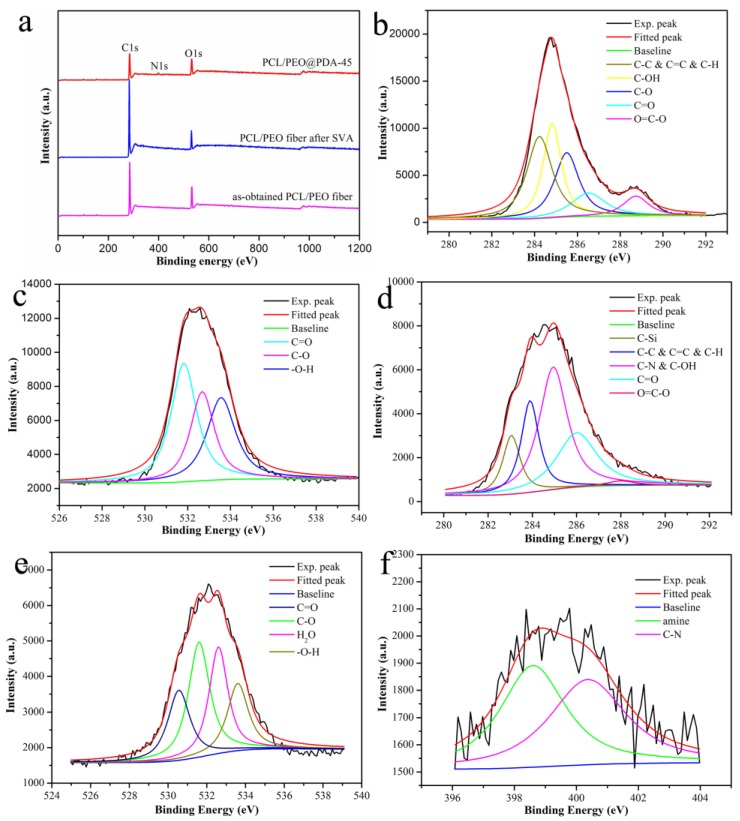
Survey XPS spectra of the fiber composites (**a**) and the peak deconvolutions: (**b**,**c**), C1s and O1s in the PCL/PEO fiber after SVA treatment, respectively; (**d**–**f**), C1s, O1s, and N1s in the PCL/PEO@PDA-45 composite, respectively.

Next, the X-ray photoelectron spectroscopy (XPS) was measured to investigate the interfacial elemental compositions of the obtained composite fiber materials. First, the survey XPS spectra of different fiber composites are shown in Figure 4a. Some characteristic peaks appeared such as general C1s and O1s as well as additional N1s peaks in the PCL/PEO@PDA-45 composite. It should be noted that the as-obtained PCL/PEO fiber showed the relative atomic ratios of C and O elements with values of 72.6% and 26.5%, respectively. After SVA treatment, the relative atomic ratios of C and O elements showed the values of 77.3% and 21.7%, respectively. In addition, the atomic ratios of C, O, and N elements for the PCL/PEO@PDA-45 composite changed with values of 73.2%, 19.6%, and 4.9%, respectively. Due to the presence of the N element and decrement of the O element, it could be reasonably speculated that the synthesized PDA particles were successfully anchored on the surface of the composite fibers. Figure 4b shows that the C1s signals of the SVA-treated PCL/PEO fiber were mainly located at 284.1, 284.8, 285.5, 286.5, and 288.7 eV, corresponding to the C–C & C=C & C–H, C–OH, C–O, C=O, and O=C–O bonds, respectively [33,38,39]. The O1s deconvolution data are shown in Figure 4c, which clarified three peaks at 531.7, 532.7, and 533.5 eV, representing the bonds of C=O, C–O, and –O–H, respectively [33,40]. In addition, Figure 4d–f illustrate the deconvolutions of the C1s, O1s, and N1s peaks in the PCL/PEO@PDA-45 composite. The C1s peak was deconvoluted to five peaks at 283.0, 283.9, 284.9, 286.0, and 288.0 eV, corresponding to the C–Si, C–C & C=C & C–H, C–N & C–OH, C=O, and O=C–O groups [26,33,38,39]. The presence of C–Si was due to the silicon plate during the testing procedure. The O1s deconvolution exhibited peaks of C=O, C–O, H_2_O, and –O–H species at positions of 530.0, 531.6, 532.6, and 533.6 eV, respectively [26,33,40]. The possible H_2_O at 532.6 eV originated from the residual moisture in the sample. The N1s deconvolution showed amine and C–N species at 398.6 eV and 400.4 eV, respectively, indicating that the PDA layer was apparently present on the fiber surface [26,37,38,41,42]. In addition, the microstructures of the SVA-treated PCL/PEO and PCL/PEO@PDA-45 samples were investigated using N_2_ adsorption–desorption isotherms. The obtained properties of the samples are generalized in Table 1. As shown in Table 1, the as-obtained PCL/PEO fiber showed the specific surface area of 8.5467 m^2^g^−1^. After SVA treatment for 45 h, the value of the specific surface area increased obviously and reached 15.3133 m^2^g^−1^, demonstrating the formation of more anchoring sites for the next modification of the PDA coating. In addition, the obtained PCL/PEO@PDA-45 after chemical modification of PDA showed a decreased BET specific surface area (9.0741 m^2^g^−1^) than the SVA-treated PCL/PEO fibers (15.3133 m^2^g^−1^), indicating that the PCL/PEO@PDA-45 composite fiber anchored numerous PDA particles in interfacial adsorption sites, facilitating the next adsorption of dye molecules. Meanwhile, the pore size and pore volume of the two samples were calculated via BJH methods. The obtained PCL/PEO@PDA-45 composite also exhibited decreased pore size and pore volume, meaning that larger pore diameters and pore volumes in the SVA-treated PCL/PEO fibers could demonstrate lots of micro/nanoscale channels for PDA nanoparticles to transfer into the composites, thereby making it effective for the next adsorption capacities experiment [43,44].

**Figure 5:**
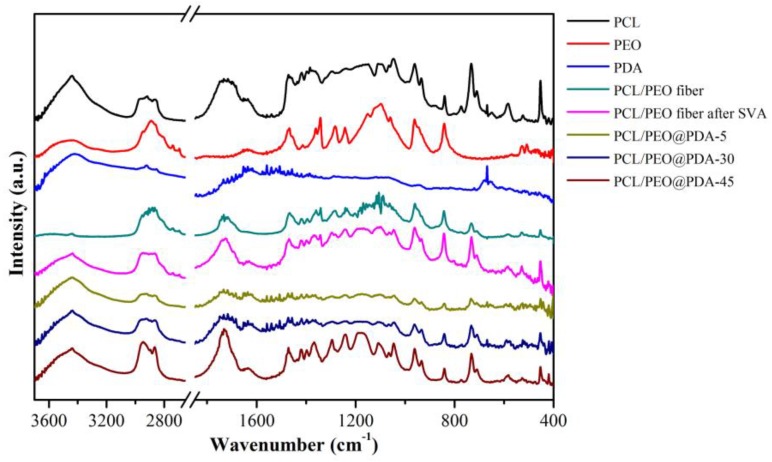
FT-IR spectra of the initial materials and the obtained composite fibers.

In order to further investigate the obtained composite fiber, the infrared curves of the initial materials and modified composites with different chemical modification time are shown in Figure 5. The infrared spectrum of the initial PEO exhibited triplet peaks of C–O–C stretching vibration at 1149, 1101, and 1060 cm^−1^ with the maximum peak at 1101 cm^−1^ [45]. Meanwhile, the characteristic peaks at 964, 1470, 2884, and 3438 cm^−1^ were derived from the initial PEO, corresponding to the rock vibration of CH_2_, the CH_2_ stretching vibration, and the terminal hydroxyl group, respectively [45,46,47,48]. As for the PDA curve, the peak at 1610 cm^−1^ was attributed to the aromatic rings stretching vibrations and the N–H bending vibrations, and the peak at 3400 cm^−1^ was attributed to the catechol –OH groups and N–H groups [30]. As shown in the spectral curves of the electrospun PCL/PEO and SVA-treated PCL/PEO, characteristic peaks at 2945, 2871 cm^−1^, and 1723 cm^−1^ were attributed to asymmetric and symmetric CH_2_ stretching vibration and ester carbonyl stretching vibration, respectively [48]. After the SVA-treated PCL/PEO was chemically modified by PDA, the obtained PCL/PEO@PDA showed new characteristic peaks at 1558, 1550, 1500 cm^−1^, corresponding to the amide bands, amines, and aromatic benzene rings in the PDA component, respectively, and accompanying the absorption peak at 1723 cm^−1^, which slightly shifted to 1730 cm^−1^ [33,45,49]. All characteristic peaks were correspondingly displayed in the infrared curve of the PCL/PEO@PDA composite. The above infrared data further demonstrates that the PDA component was successfully anchored on the surface of the substrate SVA treated PCL/PEO material.

### 3.2. Adsorption Dye Performance of PCL/PEO@PDA Composite

**Figure 6:**
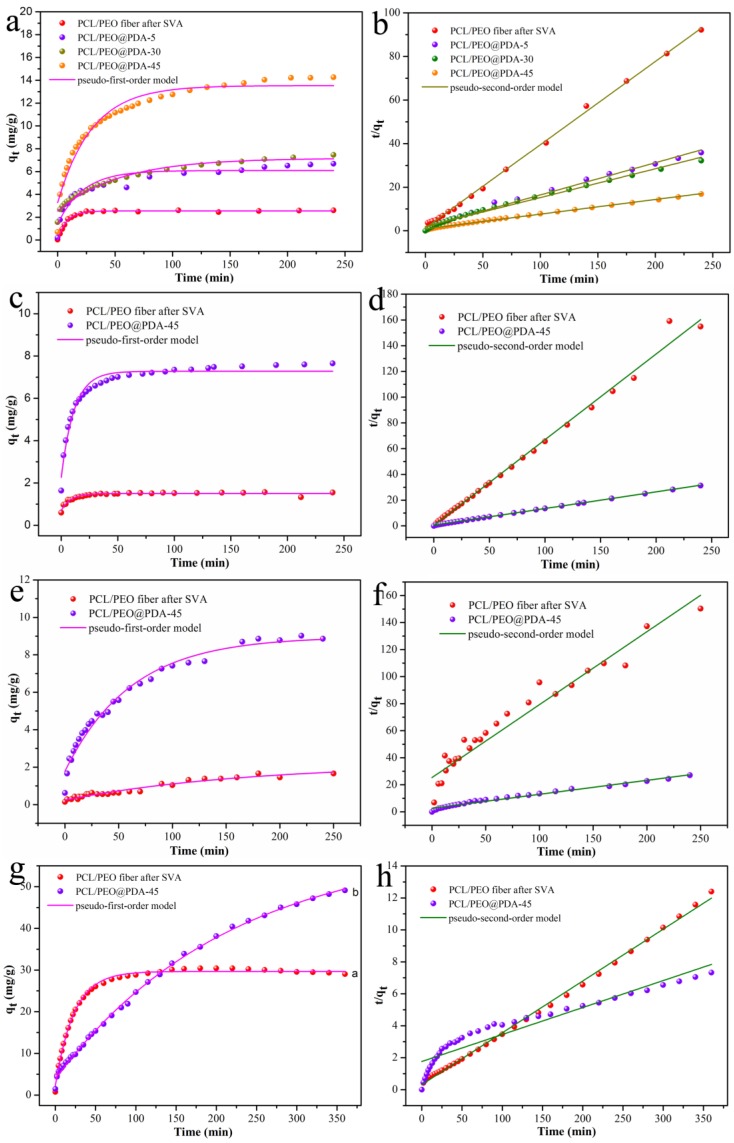
Adsorption kinetics curves of the prepared composite fibers on methylene blue (MB) (**a**,**b**), Rhodamine B (RhB) (**c**,**d**), Safranine T (ST) (**e**,**f**), and methyl orange (MO) (**g**,**h**) at 298 K.

Adsorption kinetics is of great significance in the evaluation of adsorption efficiency. A variety of adsorbents evaluated by pseudo-first-order and pseudo-second-order kinetic models have been reported, as listed in Table 2 [12,13,22,23,33,50]. The adsorption behaviors of several adsorbents towards methylene blue (MB) and methyl orange (MO) in Table 2 indicated that different adsorbents show selective adsorption properties for different dyes. For examples, magnetic cellulose beads (MCB-AC) synthesized by Luo et al. [12] showed certain adsorption capacity for MB and MO (1.8 mg/g and 1.3 mg/g, respectively), which could be well regenerated and reused. Fu et al. [13] reported the synthesis of independent PDA microspheres and the selective adsorption/separation capacities of organic dyes, where the obtained PDA microsphere adsorbent showed a better selective adsorption of MB (147.0 mg/g) than MO (almost zero). Zhou et al. [22] reported the magnetic core-shell structure of Fe_3_O_4_@PDA nanoparticles which selectively adsorbed MB (10.0 mg/g) rather than MO (2.1 mg/g) due to electrostatic attraction between dye molecules and adsorbents. Thus, both MB and MO molecules were regarded as typical model dyes for the adsorption test in present study system. The adsorption properties of our prepared PCL/PEO@PDA-45 composite and the SVA-treated PCL/PEO fiber as adsorbents for MB and MO aqueous solutions were estimated by pseudo-first-order and pseudo-second-order kinetic models. From the adsorption curves seen in Figure 6, it was found that the composite films chemically modified by PDA exhibited improved adsorption capacities than the substrate composite film. This result can be attributed to the affluent amino and hydroxyl groups on the surface of the PDA to provide more adsorption activity points for the dye molecules [33]. The following classical kinetic model equations illustrate the above adsorption mechanism:

The pseudo-first-order kinetic model Equation (1) is expressed:

(1) $$\log\;\left(q_{e}-q_{t}\right)=\;\log\;q_{e}-\frac{k_{1}}{2.303}\;t$$

The pseudo-second-order kinetic model Equation (2) is expressed as:

(2) $$\frac{t}{q_{t}}=\frac{1}{k_{2}q_{e}^{2}}+\frac{t}{q_{e}}$$

where q_e_ (mg/g) is the adsorption amount when the adsorption process reaches equilibrium; q_t_ (mg/g) is the adsorption amount when the adsorption time t (min); k_1_ (min^−1^) is the pseudo-first-order rate constants; and k_2_ (g/mg·min) is the pseudo-second-order rate constants. The obtained adsorption kinetic related data (Figure 6) are shown in Table 3. The adsorption amount (q_e_) of the dye at the equilibrium of adsorption indicated that a good fit of the adsorption experimental curves had been obtained. It can be seen from Figure 6a and Table 3 that the adsorption efficiency of the PCL/PEO@PDA adsorbent for the MB dye enhanced with the increment of the chemical modification time of PDA. In addition, the correlation coefficient R^2^ corresponding to the pseudo-first-order and pseudo-second-order kinetic model indicated that the adsorption process of the PCL/PEO@PDA-45 composite adsorbent for MO dye was more consistent with the pseudo-second-order kinetic model, while the adsorption kinetics of other adsorbents for the MB, MO, and ST dyes were more consistent with the pseudo-first-order kinetic model. It is worth mentioning that the obtained PCL/PEO@PDA-45 composite adsorbent exhibited better fitted adsorption efficiency for MO (60.22 mg/g) than that of MB (14.85 mg/g). That is to say, the chemically modified PCL/PEO@PDA-45 composite adsorbent exhibited excellent selective adsorption capacity for the anionic dye (MO) versus the cationic dye (MB) [13]. As shown in Table 2, for examples, Dong et al. [23] reported the adsorption performance of PDA/GO composite adsorbents toward various organic dyes, such as MB (1800.0 mg/g), MO (30.0 mg/g), RhB (100.0 mg/g), and neutral red (1400.0 mg/g), showing an extremely high adsorption capacity. Xing et al. [33] investigated the self-assembly of hierarchical poly(vinyl alcohol)/poly(acrylic acid)/carboxylate graphene oxide nanosheets@polydopamine (PVA/PAA/GO-COOH@PDA) composite and adsorption performances for MB (34.1 mg/g), RhB (8.4 mg/g) and Congo red (12.9 mg/g), which showed selectively MB-adsorbed process due to strong π-π stacking and electrostatic interaction. Liu et al. [50] reported that poly(catechol-tetraethylenepentamine-cyanuric chloride)@hydrocellulose(PCEC-C) composite absorbent effectively removed MO (37.2 mg/g) at 298 K due to electrostatic interaction, hydrogen bonding, and π-π stacking interaction. In our present system, to further explore the adsorption performance of the prepared composite fibers, we also studied the adsorption process of the PCL/PEO@PDA-45 composite and SVA-treated PCL/PEO fiber on typical cationic dyes (RhB and ST). The obtained results in Figure 6 and Table 3 showed that the PCL/PEO@PDA-45 composite adsorbent exhibited common adsorption properties for RhB and ST (7.7316 and 9.6628 mg/g, respectively). It is well known that most forces between adsorbents and dyes are ion interactions, electrostatic attraction, π–π stacking interactions, and host–guest interactions [13,23]. However, space steric hindrance (such as the aromatic ring of ST, and the long-chain alkyl chain of RhB) could offset the electrostatic attraction and π–π stacking interactions, and so on [50]. In addition, the Eschenmoser salt between the ortho position of the catechol phenolic hydroxyl group in PDA and the Eschenmoser structure of the dye (such as MB) assisted the 1,4-Michael addition reaction, increasing the adsorption capacity of the composite adsorbent containing a PDA component [23]. Thus, in combination with the above several adsorption systems, the PCL/PEO@PDA-45 composite adsorbent prepared in this study exhibited a selective adsorption performance on the used ion dyes. In all, the obtained PCL/PEO@PDA composite adsorbent selectively and effectively removed MO due to stronger π-π stacking and electrostatic interactions [13,23,33,50], friendly adsorbed MB because of the Eschenmoser salt formed by the 1,4-Michael addition reaction. At the same time, the PCL/PEO@PDA composite adsorbent exhibited a limited adsorption capacity on RhB and ST. The above obtained experimental results showed that present composite adsorbents could potentially be used in the wide fields of wastewater treatment.

**Figure 7:**
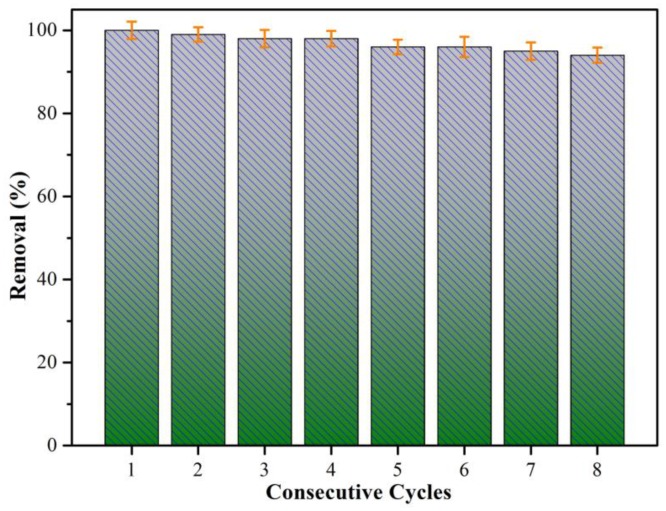
Regeneration studies of the PCL/PEO@PDA-45 composite toward MO for different consecutive cycles at 298 K.

Good stability and reusability of the adsorbent are important considerations for practical production. Adsorbents with excellent reusability have been reported in previous studies [51,52,53,54,55,56,57,58]. For example, Lu et al. [41] investigated the reuse efficiency of the PDA/PEI@PVA/PEI composite adsorbent on Ponceau S (PS) of up to 91% after ten times cycling, suggesting excellent stability and recycling ability. It should be noted that the reported PDA/PEI@PVA/PEI composite adsorbent showed an inconspicuous loss of the soluble PEI component in the adsorbent material during the adsorption measurements. Next, the adsorption stability and reusability of the PCL/PEO@PDA-45 composite was investigated. The cyclic adsorption experiment was carried out eight times in succession, and each adsorption time was the same as that in Figure 6c for four hours. The dye-adsorbed PCL/PEO@PDA composite was thoroughly washed with ultrapure water and ethanol to remove the adsorbed dye molecules and by-products generated during the adsorption process as much as possible, which was utilized for further cyclic adsorption. The cyclic adsorption efficiency of the PCL/PEO@PDA composite adsorbent towards MO dye is shown in Figure 7. The dye removal efficiency was calculated using the following equation:

(3) $$\mathrm{Removal}\%=\frac{C_{0}-C_{t}}{C_{0}}\times 100$$

After eight adsorption-desorption cycles, the removal efficiency of the PCL/PEO@PDA composite adsorbent on MO dye decreased from 99% to 93%, indicating excellent stability and recyclability of the adsorbent. The slight decrease in the removal efficiency of the PCL/PEO@PDA composite adsorbent was ascribed to the loss of trace amounts of PDA nanoparticles anchored on the adsorbent surface during the washing process or fewer residual dye molecules on the adsorbent surface [33]. It could be reasonably speculated that the possible loss of the soluble PEO component in our PCL/PEO@PDA-45 composite adsorbent showed an inconspicuous state during the adsorption measurements. The above cyclic adsorption results indicated that the PCL/PEO@PDA composite showed significant adsorption efficiency, stability, and recyclability [59,60,61,62,63,64,65].

## 4. Conclusions

In summary, we successfully designed and prepared PCL/PEO@PDA composites with significantly excellent adsorption capacity and stability. The SVA-treated PCL/PEO fibers exhibited a pleated microstructure, which increased the specific surface area of the smooth electrospun fibers and provided more active attachment sites and spaces for the subsequent chemical modification of polydopamine. The synthesized PCL/PEO@PDA composite after the chemical modification of PDA possessed numerous active sites to anchor the next dye molecules during the adsorption process. Furthermore, the PCL/PEO@PDA composite adsorbent showed a better adsorption capacity than the SVA-treated PCL/PEO composite. Simultaneously, the PCL/PEO@PDA composite adsorbent selectively adsorbed the anionic dye MO, exhibiting better adsorption efficiency than the adsorption toward the used cationic dyes (MB, RHB, and ST). In addition, the PCL/PEO@PDA composite could be separated from the dye solutions to avoid possible agglomeration, exhibiting significant regenerative and reproducible capability for dye removal. The current research work provided a new approach to designing and preparing PDA-based composite adsorbents, showing potential practical applications in the field of wastewater treatment.

## Figures and Tables

**Table 1:** Physical data of as-prepared different fiber composites.

Samples	Specific Surface Area (m^2^g^−1^)	Average Pore Diameter (nm)	Pore Volume (cm^3^g^−1^)
As-obtained PCL/PEO fiber	8.5467	24.1263	0.008736
PCL/PEO fiber after SVA	15.3133	47.0420	0.012631
PCL/PEO@PDA-45	9.0741	27.1665	0.008920

**Table 2:** Comparison of the adsorption capacity of other adsorbents in the previous studies at 298 K.

NO.	Materials	q_e_ (mg/g)		Characteristics	Ref.
		MB	MO		
1	Magnetic cellulose beads (MCB-AC)	1.8	1.3	Selective magnetic response, environmentally friendly process, reusable spherical beads.	[12]
2	PDA microspheres	147.0	almost zero	Selective adsorption of cationic dyes, economical adsorption and separation.	[13]
3	Fe_3_O_4_@PDA NPs	10.0	2.1	Selective adsorption capacity for cationic dyes, magnetic core-shell structure, easily magnetic separation.	[22]
4	PDA/GO	1800.0	30.0	Controllable PDA layer thickness, high surface area structure, excellent adsorption performance.	[23]
5	poly(vinyl alcohol)/poly(acrylic acid)/carboxylate graphene oxide@polydopamine(PVA/PAA/GO-COOH@PDA)	31.3	-	Environmentally friendly and controllable preparation method, larger specific surface area, excellent reusability.	[33]
6	poly(catechol-tetraethylenepentamine-cyanuric chloride)@hydrocellulose (PCEC-C)	-	37.2	Selective adsorption of anionic dyes, simple method and better adsorption stability.	[50]
7	PCL/PEO@PDA-45	14.8	60.2	Selective adsorption of MO, convenient and controllable method, excellent stability and reuse.	Present work

**Table 3:** Adsorption kinetic parameters of SVA treated PCL/PEO and PCL/PEO@PDA adsorbents toward dyes at 298 K.

MB	Pseudo-First-Order Model			Pseudo-Second-Order Model		
	q_e (_mg/g)	R^2^	K_1_ (min^−1^)	q_e_ (mg/g)	R^2^	K_2_ (g/mg⋅min)
PCL/PEO after SVA	2.5533	0.9949	0.1270	2.6183	0.9987	0.3809
PCL/PEO@PDA-5	6.0916	0.8968	0.0459	6.7700	0.9931	0.1457
PCL/PEO@PDA-30	7.1766	0.9738	0.0184	7.5614	0.9904	0.1297
PCL/PEO@PDA-45	13.5342	0.9576	0.0335	14.8522	0.9966	0.0669
RhB						
PCL/PEO after SVA	1.5026	0.9344	0.1217	1.5018	0.9923	1.1646
PCL/PEO@PDA-45	7.2790	0.9668	0.0878	7.7316	0.9997	0.0286
ST						
PCL/PEO after SVA	2.1838	0.9566	0.0059	1.8543	0.9358	0.0115
PCL/PEO@PDA-45	8.9755	0.9788	0.0162	9.6628	0.9826	0.0041
MO						
PCL/PEO after SVA	29.6755	0.9963	0.0432	30.8547	0.9980	0.0323
PCL/PEO@PDA-45	60.2260	0.9983	0.0046	59.2417	0.8960	0.0136
